# Experimental Manufacture of Paper for War Maps

**DOI:** 10.6028/jres.105.069

**Published:** 2000-12-01

**Authors:** Charles G. Weber, Merle B. Shaw

## Abstract

Early in World War II, a new map paper was developed that greatly improved the quality and performance of war maps. The National Bureau of Standards cooperated in the development and, subsequently, determined by experimental manufacture how to make the paper from commercially available raw materials. The best results were obtained in experimental manufacture by using fiber furnishes of 100-percent strong bleached sulfate pulps with the addition of melamine-formaldehyde resin to increase the wet strength and titanium dioxide to produce the desired capacity. It was essential that the beating be very carefully controlled to preserve the maximum fiber strength. The most critical requirements from a manufacturing standpoint were very high resistance to tear, high wet tensile strength, high opacity, and good smoothness. A moderate degree of wildness was not objectionable. The data obtained by experiments were applied to initiate the commercial production of the new paper to meet unprecedented tonnage requirements.

## I. Introduction

The Paper Section of the National Bureau of Standards cooperated actively with the Army Map Service of the Corps of Engineers in the development of a new type of map paper that proved highly important during World War II. Unique qualities built into this paper gave satisfactory performance in contact with the water, mud, and grime of the battlefield that had disintegrated papers heretofore used. This Bureau assisted in bringing the new type of paper into commerical production after its merits were established. Information essential to this step was obtained through semicommercial paper-making experiments. A detailed specification^2^ for the paper was formulated at the Army Map Service with the cooperation of the Bureau. The most important feature of the paper was its high wet strength which was obtained by the relatively new development of resin bonding.^3^ ^4^ ^5^

The requirements of the specification were very stringent. They demanded the rugged strength when dry, wet, or oil-soaked that gave the maps their durability. In addition, they called for low expansivity; high opacity when wet, dry, or oiled; good writing quality when wet or dry; low acidity; and smoothness suitable for printing multicolor line maps and 200-line photomaps. Close limitations were placed on thickness, weight, and moisture content. The paper-making experiments determined how the paper could be made from available raw materials to meet the specification.

The information obtained was applied with much success in assisting commercial mills to get into successful production of the paper with a minimum of delay. It was of great value also as a basis for judging from the properties of papers not meeting all details of the specification, changes in composition or manufacturing technique necessary to bring the papers up to standard.

When it became important to explore the possibilities of saving space and weight in the shipment of maps by air, the results were especially useful. With the data at hand, it was possible for the Paper Section to produce quickly, in quantities sufficient for printing trial lots of maps, papers embodying optimum qualities obtainable in lighter weights. The maps from these printings were used by the Army Engineers to establish the utility and durability of lightweight maps.

## II. Experimental Paper-making Equipment

The equipment of the Bureau’s paper mill is semicommercial in size, and is adapted to the experimental manufacture of papers under conditions which simulate those of industrial plants. Detailed descriptions and photographs of the equipment are contained in previous publications.^6^ ^7^ ^8^ The equipment used in this particular work consisted essentially of a 50-pound beater with copper-lined wooden tub and manganese-bronze bars and plate; a jordan refiner with bars of bronze and steel alloy; a four-plate, flat screen; a 29-inch Fourdrinier paper-making machine with a wire 33 feet long, two presses, nine 15-inch dryers, a machine calender stack of 7 rolls, and a reel; and a five-roll supercalender.

## III. Fibrous Raw Materials

The extremely high strength required for the map paper made it essential that all or a large proportion of the fibers used be of high strength. Rag fibers were not given consideration because neither an adequate supply of rags nor sufficient rag-cooking equipment was available to meet anticipated needs. Hence, the experiments were confined to commercially available bleached wood pulps. The following pulps were used either singly or in mixture:

1. Northern bleached sulfate, made by cooking eastern spruce in a strong solution containing caustic soda and sodium sulfide. This was very strong, long-fibered wood pulp.
2. Bleached sulfate No. 2 made from mixed species of relatively poor wood. This was somewhat like No. 1, but softer and weaker because of the lack of adequately selected woods at the producing mill.
3. Western bleached sulfate, a very strong wood pulp made from western hemlock. It was comparable to No. 1 in quality.
4. Bleached sulfite, made by cooking eastern spruce wood in a solution of calcium bisulfite. This was a standard quality of pulp used for bond papers. It was a relatively long-fibered, strong pulp, but not as strong as the sulfate-cooked pulps made from selected woods.
5. Deciduous wood soda, a filler pulp produced by cooking wood in a strong solution of caustic soda. This was an unusually strong soda pulp. However, no pulp of this type is comparable to the sulfate or coniferous sulfite pulps in strength or length of fibers. Its use in a paper contributes to printing quality and formation of paper rather than to the strength.
6. Deciduous wood sulfite, a short-fibered filler pulp made by cooking deciduous woods in a solution of calcium bisulfite. It was comparable to soda pulp in most of its characteristics.

The chemical characteristics of these pulps are shown in table 1.

## IV. Manufacturing Procedure

**Figure 1:**
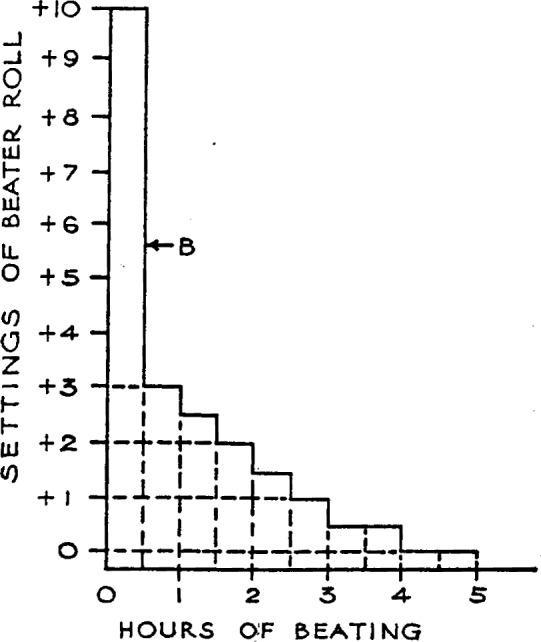
Standard practice of lowering the beater roll for all experimental map papers. “B”, Pattern of beating curve for all papers. Value for roll settings are turns of the handwheel above (+) contact (0) of roll with bedplate. One turn moves the roll 0.008 inch. Lighter bar down at start.

Before the furnish was added to the beater, the roll was raised off the plate a definite number of turns. The position of the beater roll is expressed as the number of turns above (+) or below (−) zero setting, which is the point of contact between the roll and the bedplate. One turn moves the roll 0.008 inch. The various positions of the roll and the time intervals for each during the beating of a furnish are shown in figure 1.

Forty-eight pounds of pulp was furnished to the beater in each instance. The pigment, titanium dioxide, was added during the furnishing. After the furnishing, which took about 15 minutes, the beater roll was gradually lowered by definite steps at fixed intervals throughout the beating, as indicated by the beating curve in figure 1. The rosin size was added 1 hour, and the alum (aluminum sulfate) ½ hour, before completion of the beating cycle.

The beaten stock was dropped to a chest and pumped in a continuous stream through the stuff box and the jordan to the paper machine without the use of a machine chest. The stuff box was of the familiar regulating type, having a constant head over an adjustable orifice. Screen plates with 0.018-inch slots were used for all runs. The jordan was used as a mixer only, on the same setting for all runs.

When melamine-formaldehyde resin was used it was added as a colloidal solution made by dissolving the resin in the form of a fine white powder in warm water acidified with hydrochloric acid. The solution was added continuously to the stock leaving the screen, just before it entered the head box of the paper machine. The stock was uniformly mixed by baffles in the head box, and the temperature of the stock was maintained at 90° ±2° F at that point.

Rosin size was used in all papers. It was precipitated with papermakers alum, Al_2_(SO_4_)_3_, which was used to control the pH of the stock. The papers were opacified with titanium dioxide. The finish imparted by the small machine calender was insufficient, and it was necessary to improve the smoothness by light supercalendering.

## V. Testing

All physical and chemical tests of the pulps and papers referred to in this article were made in accordance with the official methods^9^ of the Technical Association of the Pulp and Paper Industry. Wet bursting strength and wet tensile strength were determined on samples immediately after immersion for 1 hour in water at 73° F, using the same procedure as for corresponding tests on samples conditioned in the normal manner. The pH of mill waters at the beater, stuff box, and head box was determined electrometrically, using a quinhydrone electrode.

## VI. Results

**Figure 2:**
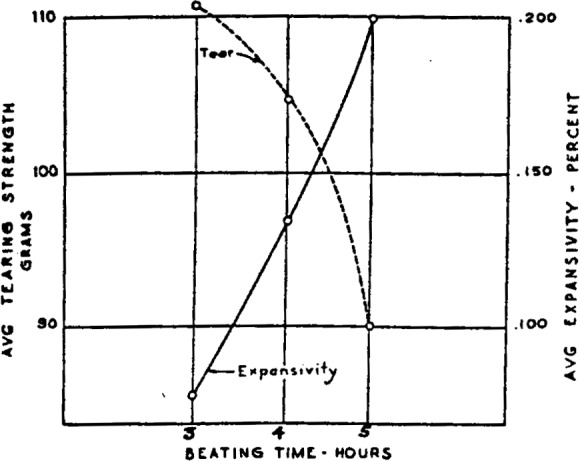
Relationship between degree of beating and expansivity and tearing strength of experimental map papers.

The first papers were made of all eastern sulfate. It was the strongest bleached commercial wood pulp available at the time, and the first problem was to determine how to make the best possible paper from it. By making a series of papers, varying one factor at a time, it was established that the most suitable map paper produced was made with only 3 hours of beating with the jordan set for mixing only. The sheet had a “wild” formation^10^ when judged by the time-honored method of looking through it. However, visible formation *per se* was of no importance, and no reference was made to it in the specification. Tearing strength and wet tensile strength in the cross direction were the most critical properties from the control standpoint. More than 3 houses of beating resulted in low tearing strength and correspondingly high expansion. This relationship is shown graphically in figure 2. This control of beating was absolutely essential to the production of a paper with satisfactorily high resistance to tear and low distortion.

High wet strength and important increase in the dry or normal strength was obtained by using melamine-formaldehyde resin. Three percent was required to obtain the necessary wet strength, and more than 3 percent reduced the resistance to tear rather sharply by a continuous film effect.

Some difficulty was encountered in obtaining reasonably high retention of the opacifying pigment when the melamine resin was first used. A control paper without this resin, with 3 percent of titanium dioxide added, had an opacity value of 93 percent. The next paper made was a duplicate, except that 3 percent of melamine resin was added. Less alum was required to adjust the pH because the resin solution added was strongly acid. Poor retention of pigment resulted, and the opacity dropped to 86 percent. The loss of pigment was found to be caused by a deficiency of alumina present from hydrolysis of the alum. It was corrected by adding an excess of alum and then adjusting the pH with sodium carbonate. Apparently, the important role in holding the particles of pigment to the fibers is played by positively charged alumina, which acts as an electrostatic cement between the fibers and the pigment, each of which carry negative charges. The use of sodium phosphoaluminate as a source of alumina, which does not reduce pH, had little effect on retention. However, by adding an excess of alum, excellent opacity was again obtained. Thereafter, 3 percent of alum, plus sufficient sodium carbonate to give a pH of 4.5 at the head box, was used in all instances. These results are in complete agreement with the published findings of Martin and Willets 11 regarding the influence of the positively charged alumina on the retention of fillers in paper.

**Figure 3:**
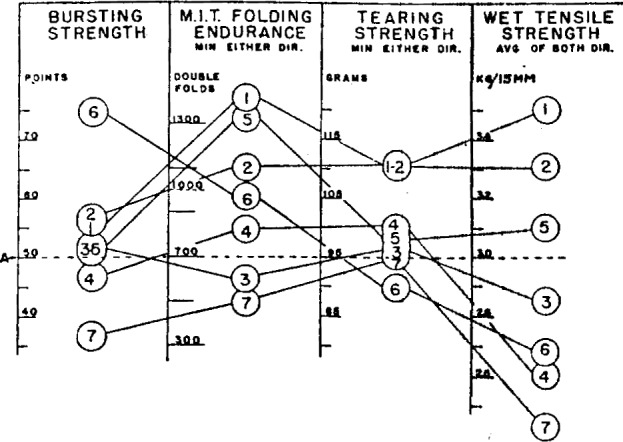
Relationship between fiber furnish and some strength properties of experimental map papers. *A*, specification level. 1. 100% northern bleached sulfate. 2. 100% western bleached sulfate. 3. 87½% northern bleached sulfate, 12½% deciduous sulfite. 4. 87½% northern bleached sulfate, 12½% strong soda. 5. 90% northern bleached sulfate, 10% bleached sulfite. 6. 70% northern bleached sulfate, 30% bleached sulfite. 7. 100% sulfate from mixed species. The beating time for all papers was 3 hours. 3% melamine resin added.

It was found possible to meet all of the requirements of the specification by use of 100 percent of either eastern sulfate or western sulfate. However, attempts to obtain satisfactory strength when using the sulfate made from mixed species of wood of poor quality were unsuccessful. Apparently the presence of short, soft fibers from deciduous woods reduced the fiber strength below the minimum required for this quality of paper. Figure 3 shows a comparison of some of the most important properties of the best papers that could be made by using various fiber furnishes. Attempts to extend the strong, and somewhat critical, sulfate pulps by the addition of filler pulps were only partially successful. The addition of as little as 12½ percent of deciduous wood sulfite or soda, both high-grade filler pulps, resulted in failure to meet the specification in some respects. Even coniferous sulfite, which is a relatively strong wood pulp, could not be used in amounts over about 10 percent without failure to meet the required tearing strength.

## VII. Application to Commercial Production

With the information developed in the experimental work, it was possible to give maximum assistance in extending the commercial manufacture of the paper to widely distributed mills to meet quickly the unprecedented needs of the armed forces. Technical service was provided for mills on their initial orders and more than a dozen mills were able quickly to make paper meeting the very high standards of the armed forces. Table 2 contains pertinent data on the properties of nine map papers made early in the program at widely distributed commercial mills. All these mills followed specific technical instructions from the Bureau based on information obtained in the experimental work. The success in applying the information is shown by the consistent manner in which the various papers conformed to, and in many instances exceeded, the most difficult requirements of the specification. Production exceeding 10,000,000 pounds per month within approximately 6 months after initiation of commercial manufacture of an entirely new type of paper was accomplished with a minimum of delay or loss of critical raw materials.

That these papers were well made is attested by the following statement in a letter of commendation from the Office of Chief of Engineers, War Department, “Millions of maps have been printed on this high wet-strength paper, and their superior durable qualities have proved eminently satisfactory to troops in all theaters of war.”

## VIII. Lightweight Paper

Late in the war, the Army Map Service investigated the possibility of reducing the shipping weight and bulk of maps. To aid in this work, the Bureau made a number of experimental runs and determined the qualities that could be obtained in reduced weights. The Map Service was furnished with sufficient quantities of two experimental papers, 12½ percent and 25 percent below the standard weight, respectively, for printing and performance tests. When the performance of the lighter paper was found to be sufficiently promising to justify commercial-scale purchase, the Paper Section had the data for a detailed specification. This paper was composed of 100 percent northern or western bleached sulfate with 3 percent of melamine resin for wet strength and 3 percent of titanium dioxide pigment for opacity. It was made with absolute minimum of beating to preserve the fiber strength. The resulting tendency to “wildness” in formation did not affect adversely the printing characteristics or appearance of the finished maps.

## IX. Summary and Conclusions

The development of the improved paper for war maps and the commercial manufacture of it were accomplished through cooperative effort. The Bureau was particularly active in determining how the paper should be made from available materials for optimum performance.

The best results were obtained in experimental manufacture by using fiber furnishes of 100-percent strong bleached sulfate pulps with melamine-formaldehyde resin for wet strength and titanium dioxide for opacity. It was essential that the beating be very carefully controlled to preserve the maximum fiber strength. The most critical requirements from a manufacturing standpoint were the very high resistance to tear, high wet tensile strength, high opacity, and good smoothness. A moderate degree of wildness was not objectionable.

A lightweight paper was developed that reduced the shipping weight and bulk of maps by 25 percent. It was composed of 100 percent of the strongest bleached fibers with 3 percent of melamine resin and 3 percent of titanium dioxide. It was made with absolute minimum of beating.

Washington, September 17, 1946.

## Figures and Tables

**Table 1:** Chemical characteristics of fibrous materials used in experimental manufacture of war-map papers

Pulp	Alpha-cellulose content^1^	Beta-cellulose content^1^	Gamma-cellulose content^1^	Pentosans	Copper number	Ash^2^	Resin^2^	Acidity of pulp (glass-electrode method)	
								Cold-water extraction	Hot-water extraction

	%	%	%	%		%	%	pH	pH
1. “Northern” bleached sulfate	85.4	8.6	6.0	6.5	0.7	0.1	0.1	5.8	5.3
2. No. 2 bleached sulfate	83.8	10.2	6.0	8.1	1.9	.3	.1	6.1	5.5
3. “Western” bleached sulfate	86.3	6.3	7.4	5.2	.9	.2	.1	6.7	6.2
4. Coniferous wood sulfite	84.4	3.6	12.0	4.2	1.3	.2	.4	5.8	5.5
5. Deciduous wood soda	84.6	13.1	2.3	15.3	1.3	3.0	.4	4.2	3.6
6. Deciduous wood sulfate	83.6	10.9	5.5	6.0	1.0	.2	.1	6.9	6.2

1 Based on total cellulose.

2 On oven-dry basis.

**Table 2:** Test data on commercial, wet-strength map papers from nine different mills of wide geographic distribution

Property	Manufacture									Specification requirements
	A	B	C	D	E	F	G	H	I	

Weight, 17×22: 1,000,lb	49.0	47.7	48.7	49.2	49.1	48.8	47.7	47.1	46.6	48±10%.
Thickness, inch	0.0043	0.0043	0.0043	0.0045	0.0044	0.0043	0.0043	0.0042	0.0043	0.0040±10%.
Bursting strength, points	64	55	55	69	55	54	47	51	55	50 minimum.
Bursting strength, wet, points	30	23	24	35	29	29	27	24	24	20 minimum.
Tearing strength, grams:										
Machine direction	95	95	100	103	101	93	106	104	100	95 minimum.
Cross direction	110	107	109	121	130	101	118	122	109	Do.
Tensile strength, wet, kg/15mm:										
Machine direction	4.4	3.9	3.9	4.5	3.6	3.7	4.4	4.9	3.9	3.5 minimum.
Cross direction	2.9	2.6	2.5	2.9	2.5	2.5	2.6	2.5	2.5	2.5 minimum.
Folding endurance (MIT), double folds:										
Machine direction	1,590	1,070	1,460	1,355	1,040	1,330	1,280	1,240	1,460	700 minimum.
Cross direction	940	805	720	1,295	1,050	1,440	740	770	720	Do.
Smoothness (Beck), seconds	63	53	46	37	43	42	55	51	46	50 to 100.
Opacity, percent	91	92	93	93	90	90	91	90	93	91 minimum.

## Footnotes

2 Specification No. 43-142, Army Map Service, Wash., D. C.

3 C. G. Weber, Notes on resin-bonded, wet-strength papers, Printing Equip. Eng. 70, No. 5: 38 (Aug. 1945).

4 Geo. E. From, Melamine resins for development of wet and dry strengths of paper, Pulp & Paper Mag. (Canada) 46, No. 3: 157 (March 1945).

5 Ralph W. Kumler, Melamine resins in industry, So. Pulp *&* Paper J. 19, No. 5: 20 (May 1946).

6 M. B. Shaw and G. W. Bicking. Caroa fiber as s papermaking material, Tech. Pap BS 21. 338 (1927) T340.

7 M. B. Shaw and G. W. Bicking. Further experimental production of currency paper in the Bureau of Standards paper mill, BS J. Research 3, 904 (1929) RP 121.

8 M. B. Shaw. Equipment and research work of the Bureau of Standards paper mill, Paper Trade J. 89. No. 19, 60 (1929).

9 Copies of the Methods available from tbe Association at 122 E. Forty-Second Street. New York 17, N. Y.

10 A paper is said to have wild formation when the fibers are so distributed as to present a mottled, streaked, or “curdled-milk” appearance when viewed against light.

11 S. W. Martin and W. R. Willets, Retention of fillers, Tech. Assn. Papers, Series XXIII, No. 1, 294 (1940). Tech. Assn. of the Pulp *&* Paper Ind.

